# Effects of production and market factors on ethanol profitability for an integrated first and second generation ethanol plant using the whole sugarcane as feedstock

**DOI:** 10.1186/1754-6834-7-26

**Published:** 2014-02-21

**Authors:** Stefano Macrelli, Mats Galbe, Ola Wallberg

**Affiliations:** 1 Department of Chemical Engineering, Lund University, PO Box 124, SE-221 00 Lund, Sweden

**Keywords:** Cellulosic ethanol, Second generation ethanol, Techno-economic analysis, Sensitivity analysis, Process integration, Process simulation, Minimum ethanol selling price, Production cost, Sugarcane

## Abstract

**Background:**

Sugarcane is an attractive feedstock for ethanol production, especially if the lignocellulosic fraction can also be treated in second generation (2G) ethanol plants. However, the profitability of 2G ethanol is affected by the processing conditions, operating costs and market prices. This study focuses on the minimum ethanol selling price (MESP) and maximum profitability of ethanol production in an integrated first and second generation (1G + 2G) sugarcane-to-ethanol plant. The feedstock used was sugarcane juice, bagasse and leaves. The lignocellulosic fraction was hydrolysed with enzymes. Yields were assumed to be 95% of the theoretical for each of the critical steps in the process (steam pretreatment, enzymatic hydrolysis (EH), fermentation, solid/liquid separation, anaerobic digestion) in order to obtain the best conditions possible for ethanol production, to assess the lowest production costs. Techno-economic analysis was performed for various combinations of process options (for example use of pentoses, addition of leaves), EH conditions (water-insoluble solids (WIS) and residence time), operating cost (enzymes) and market factors (wholesale prices of electricity and ethanol, cost of the feedstock).

**Results:**

The greatest reduction in 2G MESP was achieved when using the pentoses for the production of ethanol rather than biogas. This was followed, in decreasing order, by higher enzymatic hydrolysis efficiency (EHE), by increasing the WIS to 30% and by a short residence time (48 hours) in the EH. The addition of leaves was found to have a slightly negative impact on 1G + 2G MESP, but the effect on 2G MESP was negligible. Sugarcane price significantly affected 1G + 2G MESP, while the price of leaves had a much lower impact. Net present value (NPV) analysis of the most interesting case showed that integrated 1G + 2G ethanol production including leaves could be more profitable than 1G ethanol, despite the fact that the MESP was higher than in 1G ethanol production.

**Conclusions:**

A combined 1G + 2G ethanol plant could potentially outperform a 1G plant in terms of NPV, depending on market wholesale prices of ethanol and electricity. Therefore, although it is more expensive than 1G ethanol production, 2G ethanol production can make the integrated 1G + 2G process more profitable.

## Background

The need to produce cheap renewable fuels to replace fossil fuels is reflected in the political agendas of many countries, aimed at the development of a reliable energy source to ensure fuel security, promote rural development and to address climate change by reducing greenhouse gases emission [1-5]. Among the alternative biofuels, ethanol from sugarcane can provide a substantial contribution in terms of the amount produced and the environmental impact, especially if the lignocellulosic fraction of the sugarcane is also used for fuel production [6-9]. Indeed, high volumes of second generation (2G) ethanol can be produced from sugarcane bagasse and leaves, which are the residues of the current sugarcane-to-ethanol industry. 2G technology is not as mature as first generation (1G) ethanol production, and is thus less economically feasible. However, some companies have set out to demonstrate its feasibility through the construction of commercial-scale plants [10-12]. The availability of sugarcane for ethanol production is affected by volatility on the world market due, in part, to the demand for this raw material to make sugar for the food industry [13,14]. Bagasse and leaves are also combusted to generate bioelectricity, especially in areas where there are no other means of generating electricity, or only seasonably available sources. For instance, in Brazil, hydropower is the main source of electricity, while sugarcane residues can provide a suitable complement during the dry season [15-17]. Moreover, biorefineries producing alternative and/or more profitable commodities than ethanol from sugar- and lignin-containing materials may reduce the long-term profitability of ethanol plants due to raw material competition [18,19].

Market prices and competition for feedstock are the forces driving the interest in technologies that can reduce costs of ethanol production. The production cost of bioethanol can be reduced by resolving technical problems, maximizing the yield and productivity, and optimizing the process design on a larger scale [16,20]. The ethanol yield from sugarcane can be increased if the bagasse, already present on-site as 1G process waste, is treated to produce 2G ethanol, instead of being combusted to produce electricity, although the profitability is strongly dependent on the wholesale price of ethanol. Sugarcane crushing and fermentation of the readily available sugars are the main process steps in the production of 1G ethanol. However, the production of 2G ethanol is more complex, as the lignocellulosic material must undergo pretreatment to break down the structural matrix, and the polymeric sugars must be hydrolysed to fermentable monomers before microbial conversion to ethanol can take place. The main challenges in this process are to maximize the conversion of lignocellulose and minimize the loss of sugars, which is difficult using mild operating conditions [21]. Dry leaves are one of the residues of the sugarcane harvest, together with the tops and green leaves, and it is current practice to either burn them or leave them on the field as fertilizer and for pest control. Including the dry leaves in the process could further increase the ethanol output. It has been found that about 65% of the residue from harvesting can be removed as dry leaves from the fields without causing negative agricultural effects [22].

One efficient way to produce 2G ethanol from sugarcane bagasse and leaves is steam pretreatment followed by enzymatic hydrolysis (EH) and ethanol fermentation carried out by yeast [23]. However, pretreatment also generates by-products due to lignocellulose degradation (furans, aldehydes, phenolics), which may inhibit hydrolysis and fermentation [24]. Several studies have reported optimal experimental conditions for pretreatment indicating a trade-off between high sugar yield and low inhibitor formation, which can be translated into high yield in hydrolysis and good fermentability [25-27]. The strategies adopted include the use of robust or genetically modified microorganisms, optimization of the amount of water-insoluble solids (WIS) loaded into the EH tanks and carefully designed process layouts. The optimal WIS content involves a trade-off between low costs and high sugar losses: at high WIS loading the capital cost and the energy required for distillation are low, but the sugar yields and their rate of release are reduced, due to low mass transfer and enzyme inhibition [28]. Several authors have reported difficulties at high WIS loading [29-31].

Furthermore, to maximize the ethanol output per ton dry sugarcane and to reduce costs, sugar fermentation must be fast and efficient, and pentose sugars, such as xylose and arabinose, which are traditionally slowly and only partially metabolized to ethanol, must be converted [32-34]. Instead of trying to ferment the pentoses to produce ethanol, they can be used in biogas production via anaerobic digestion, and the biogas can be combusted to provide electricity. However, this is not always economically feasible, as the derived income is not sufficient to cover the capital cost of the equipment, and it depends on internal factors such as pentose concentration and biogas productivity, as well as external factors, for example wholesale prices of electricity and ethanol [20].

**Figure 1:**
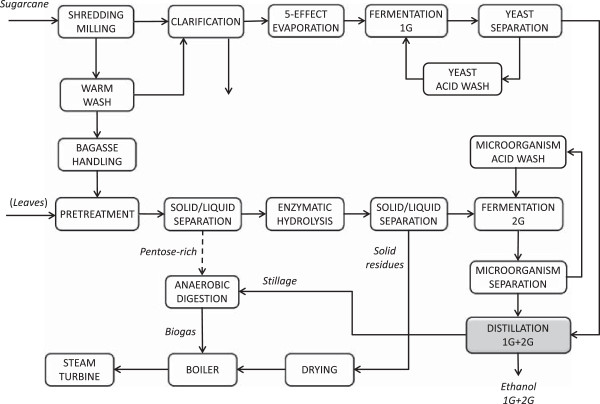
**Layout for the integrated 1G + 2G ethanol plant.** 1G, first generation; 2G, second generation.

Studies on sugarcane ethanol distilleries have found that energy can be saved and extra electricity generated by including a drying unit to efficiently remove moisture from the fuels (bagasse, leaves, EH solid residues) prior to combustion, by increasing the boiler pressure to raise the enthalpy of the steam entering the turbines, and by integrating and optimizing the heat exchanger network to minimize the steam requirement for the process [20,35-38]. The advantages are greater when 1G and 2G ethanol processes are integrated as energy and material streams are shared within the same plant. In an autonomous distillery the traditional process to produce 1G ethanol is divided into multiple stages where the sugar is first extracted from sugarcane by milling. The juice obtained is then clarified, concentrated by evaporation and fermented to ethanol, which is eventually distilled. After sugar extraction, bagasse can be used for 2G ethanol production in a 2G plant annexed to the autonomous distillery and many integration options between the processes can be envisioned due to the similarity of the transformation steps. In the 2G process bagasse can be pretreated by high-pressure steam and then the liquid fraction rich in pentoses can be separated for biogas production. The solid fraction can be treated according to the separate hydrolysis and fermentation (SHF) approach to obtain 2G ethanol from fermentable sugar monomers using either the same strain as in 1G fermentation or a different microorganism. As a result, the integration of 1G and 2G processes by stream mixing is performed with the aim to decrease heat demand and capital cost especially of the distillation unit. It has been shown that mixing streams from 1G and 2G ethanol plants in different steps (for example evaporation and distillation) reduces the overall ethanol production cost compared to the case when the two plants were run separately [20,39,40]. When the fermented juice is mixed with the fermented liquid from EH, the integration takes place at the distillation unit (Figure 1) and this configuration showed the advantages of having overall good performance using a simplified process configuration with minimum unit operations [20].

In addition to technical and process design considerations, the price of feedstock and enzymes, and the wholesale price of electricity and ethanol also determine the economic feasibility of an ethanol plant. The cost of enzymes has been found to have the greatest effect on the cost of 2G bioethanol production, followed by the electricity selling price and, to a lesser extent, the cost of transportation of leaves [20]. Although it has been found that doubling the enzyme dosage led to a lower 2G production cost, the total cost of using enzymes is a combination of the enzyme cost, the hydrolytic efficiency of the proteins and the effectiveness of pretreatment [20].

The production cost of 2G ethanol is affected by both process design and many variables, and the aim of this study was to determine which have the greatest effect on production cost. A techno-economic analysis was performed to assess the effects of both WIS content and residence time in the EH, addition of leaves, co-fermentation of pentoses, and enzymatic hydrolysis efficiency (EHE). The yields in the most critical conversion steps (pretreatment, EH, fermentation, solid/liquid separation, anaerobic digestion) are assumed to be close to the theoretical values in order to achieve as low a production cost as possible for each case, in order to determine which combinations of variables and strategy have the best potential to reduce the production cost. Sensitivity analysis was used to determine the maximum profitability achievable for ethanol production from the integrated 1G + 2G ethanol plant, and to compare it to that of 1G ethanol with regard to the wholesale prices of electricity and ethanol. The results of this study also provide some insight into which strategy should be adopted to achieve the greatest economic benefit.

## Results and discussion

The minimum ethanol selling price (MESP) was evaluated for an integrated sugarcane-to-ethanol plant (Figure 1) by the systematic combination of the main factors included in Table 1, yielding 48 cases. The most interesting cases were chosen for further analysis regarding profitability and variation in market prices. The operating conditions for a case or a series of cases are given in curly brackets, according to the definitions specified in Table 1.

**Table 1:** Type and values of design options, variables and cost factors

**Factors**	**Alternatives**
**Design options**	
Feedstock	Bagasse {B}, bagasse with addition of leaves {B + L}
Pentose utilization	Ethanol fermentation {C5 EtOH} Biogas production {C5 biogas}
**Design variables**	
EH residence time	48 hours {48 h}, 96 hours {96 h}
WIS loading in EH	10% {10% WIS}, 20% {20% WIS}, 30% {30% WIS}
**Cost factors**	
Enzymatic hydrolysis efficiency	100% {100% EHE}, 250% {250% EHE}
Electricity selling price^a^	43, 87^b^, 140 US$/MWh
Wholesale ethanol price^a^	0.3, 0.6^c^, 1.0 US$/L
Sugarcane cost	30, 65^b^, 100 US$/dry ton
Leaves cost	13, 26^b^, 39 US$/dry ton

^a^Producer selling price of the commodity at the gate of the plant; ^b^personal communication with experts from Centro de Tecnologia Canavieira, Piracicaba, Brazil; ^c^calculation based on data from CEPEA [56]. EH, enzymatic hydrolysis; EHE, enzymatic hydrolysis efficiency; WIS, water-insoluble solids.

The case investigated in this work was based on a process design previously modelled where the stream integration between 1G and 2G plants occurred by mixing the fermented broths prior to the distillation unit (Figure 1) [20]. The most relevant advantages of this plant configuration are the full bagasse throughput for 2G ethanol, yielding the highest ethanol production volume per ton dry sugarcane, and the low investment cost, due to the simplified configuration.

The assumption of a 95% yield in each fundamental conversion step (pretreatment, EH, fermentation, solid/liquid separation, anaerobic digestion) may appear unrealistic, especially when simulating challenging conditions, for example the {C5 EtOH, 30% WIS, 48 h} case. Therefore, such similar cases in the sensitivity analysis, which have not been validated experimentally, should only be considered for illustrative purposes. However, nearly theoretical yields can be used to estimate the potentially lowest 2G MESP attainable for each case compared to when lower yields are actually achieved. High yields could be obtained in hydrolysis and fermentation using a long residence period, improved technology, or a combination of both. Among the conversion steps involved, the pretreatment step determines a wide range of sugar losses, as the amount of sugar degradation products varies between 3% and 25% [41-44], but a recovery yield of 95% could be attained already today using suitable conditions. However, the conditions prevailing during pretreatment influence the release of sugars and the rate of EH. At high WIS loading in the EH it is generally difficult to obtain high sugar yields, but a successful experiment was described by Cannella *et al*. [45], in which the final overall sugar-to-ethanol yield approached 90%. Productivity must also be considered. In a study by Benjamin *et al*. a residence time of 72 hours was required to obtain a 90% EH yield from glucose [46]. In the SHF configuration, the EH liquid fraction, which is rich in sugars, is separated from the solid fraction and is then fermented by yeasts. A washing step is included to minimize sugar losses, which can permit to achieve a sugar recovery up to 99%, depending on the amount of water and the temperature used for washing [47], but in our study a yield of 95% was adopted in the mass balance calculations. The yield from co-fermentation assumed in these simulations was 95% for both hexoses and pentoses, and is not dependent on either the strain (yeast/bacteria) or on the concentration in the fermentation broth, which can be increased to the desired value by recirculation of the fermenting organism. Ethanol yield above 90% of the theoretical on glucose and xylose has already been achieved at low yeast concentration [48]. High ethanol productivity during co-fermentation of pentoses and hexoses is also a challenge. Experimental results obtained from co-fermentation indicate that an ethanol productivity of 2.3 g/g of dry cell per hour and about 90% yield are already feasible [49], while the productivity assumed in the simulations (15 g/L/h) could be achieved in this case using about 7 g of dry cells per litre. In anaerobic digestion, the chemical oxygen demand (COD) removal, the biogas yield and the production rate may be limited by the presence of ions, and toxic and recalcitrant compounds. Thus, 95% may be a rather optimistic figure for the conversion of organic compounds to biogas (kg biogas/kg COD), as well as methane yield for sugars and organic acids (kg CH_4_/kg CH_4_ theoretical). In a study by España-Gamboa *et al*. the COD removal was found to be 69% and the methane yield 75% of the theoretical for vinasse treatment [50]. Moreover, the highest methane yield from the liquid fraction after bagasse pretreatment has been reported to be close to 52% [20,51]. For other vinasses (from wine), both the COD removal and the methane yield were as high as those assumed in this study [52]. The COD removal rate was set to 1 g/L/h in the simulations, which is comparable to the results reported by Sanchez Riera *et al*. on biogas production from sugarcane ethanol stillage [53].

### 2G MESP

#### Use of pentoses: ethanol versus biogas production

**Figure 2:**
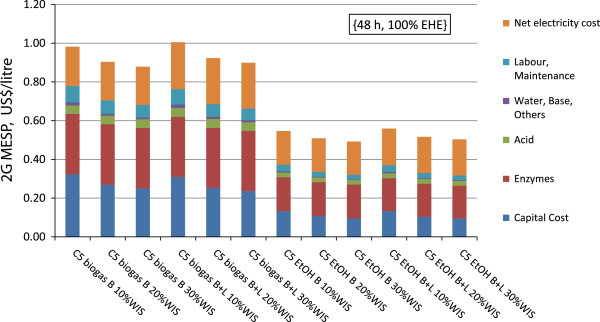
**Results for 2G MESP for the {48 h, 100% EHE} cases.** 2G MESP is presented as sum of cost items and as function of pentose utilization, feedstock and WIS. For case definitions see Table 1. 2G, second generation; B, bagasse; B + L, bagasse with addition of leaves; C5 biogas, biogas production from pentoses; C5 EtOH, pentose fermentation to ethanol; EHE, enzymatic hydrolysis efficiency; MESP, minimum ethanol selling price; WIS, water-insoluble solids.

Among the process options and variables studied, pentose utilization had the greatest impact on the 2G MESP. Ethanol and biogas production from pentoses are the two alternatives, and resulted in two clearly separated macro-regions of results regarding the 2G MESP, as shown in Figure 2 for the 48-hour EH residence time. The production of ethanol from pentoses gave the lowest 2G MESP (Table 2), ranging from 0.50 to 0.63 US$/L, but when pentoses were instead used to produce biogas, the 2G MESP increased to the range 0.88 to 1.14 US$/L. Within these two macro-regions, the other factors studied (the cost of enzymes, the addition of leaves, the WIS loading, the EH residence time) had smaller effects.

**Table 2:** 2G cost items and 2G MESP for all cases in US$/L

	**C5 biogas**						**C5 EtOH**					
**Cost items for 2G MESP**	**B**			**B + L**			**B**			**B + L**		
	**10% WIS**	**20% WIS**	**30% WIS**	**10% WIS**	**20% WIS**	**30% WIS**	**10% WIS**	**20% WIS**	**30% WIS**	**10% WIS**	**20% WIS**	**30% WIS**
Capital cost, 48 h	0.41	0.33	0.31	0.37	0.31	0.29	0.17	0.14	0.13	0.16	0.13	0.12
Capital cost, 96 h	0.50	0.39	0.35	0.46	0.33	0.31	0.22	0.17	0.15	0.21	0.16	0.14
Labour, maintenance, 48 h	0.11	0.09	0.08	0.10	0.08	0.08	0.05	0.04	0.03	0.04	0.03	0.03
Labour, maintenance, 96 h	0.14	0.10	0.09	0.12	0.09	0.08	0.06	0.04	0.04	0.06	0.04	0.04
Net electricity cost, 48 h	0.12	0.11	0.11	0.17	0.16	0.16	0.13	0.14	0.14	0.15	0.15	0.15
Net electricity cost, 96 h	0.13	0.12	0.12	0.18	0.17	0.17	0.13	0.14	0.14	0.16	0.16	0.16
Enzymes, 100% EHE	0.31	0.31	0.31	0.31	0.31	0.31	0.17	0.17	0.17	0.17	0.17	0.17
Enzymes, 250% EHE	0.12	0.12	0.12	0.12	0.12	0.12	0.07	0.07	0.07	0.07	0.07	0.07
Acid	0.04	0.04	0.04	0.04	0.04	0.04	0.02	0.02	0.02	0.02	0.02	0.02
Base	0.007	0.007	0.007	0.008	0.008	0.008	0.004	0.004	0.004	0.004	0.004	0.004
Water consumption	0.009	0.009	0.005	0.005	0.003	0.003	0.009	0.009	0.005	0.005	0.003	0.003
**2G MESP {48 h, 100% EHE}**	1.01	0.91	0.88	1.01	0.92	0.90	0.55	0.53	0.50	0.56	0.51	0.50
**2G MESP {96 h, 100% EHE}**	1.14	0.99	0.94	1.14	0.96	0.93	0.63	0.57	0.54	0.63	0.56	0.54
**2G MESP {48 h, 250% EHE}**	0.82	0.71	0.69	0.82	0.73	0.70	0.45	0.42	0.39	0.45	0.41	0.40
**2G MESP {96 h, 250% EHE}**	0.95	0.79	0.75	0.95	0.77	0.74	0.52	0.45	0.43	0.52	0.45	0.43

For case definitions see Table 1. 2G, second generation; B, bagasse; B + L, bagasse with addition of leaves; C5 biogas, biogas production from pentoses; C5 EtOH, pentose fermentation to ethanol; EHE, enzymatic hydrolysis efficiency; MESP, minimum ethanol selling price; WIS, water-insoluble solids.

The most relevant differences between the biogas-oriented configuration and the ethanol-oriented case are the additional tanks needed for anaerobic digestion of the pentoses, the additional filter presses and a larger combined heat and power (CHP) plant, which is required because of the co-combustion of a higher amount of biogas in addition to the EH solid residues. The number of tanks is dictated by the biogas productivity, which is assumed to be constant, and the organic loading rate (ton COD/h), which increases significantly when pentose sugars are digested. For the {10% WIS, 96 h} case the total investment cost increased from 408 to 464 million US$ when bagasse was the only feedstock, and from 521 to 586 million US$ when leaves were also added. The corresponding increases in capital cost, in terms of 2G MESP were 0.22 US$/L for the {B} case and 0.19 US$/L for the {B + L} case when the production of biogas from pentoses was included.

The reduction in the 2G MESP is predominantly due to the reason that the volume of 2G ethanol was used as the allocation base for costs. In fact, all variable, fixed and opportunity costs are allocated based on the 2G ethanol production volume; thus, the higher the 2G ethanol production volume, the lower the 2G MESP. The cost of electricity, enzymes and the capital cost account for 86% of the overall 2G MESP, and they are thus more sensitive to the ethanol production volume.

In spite of the revenue from the electricity export associated with biogas production from pentoses, which increased on average by 26 MW_el_ for the {B} case and by 39 MW_el_ for the {B + L} case, the electricity opportunity cost item per litre of 2G ethanol was comparable to that when the pentoses were fermented to ethanol: 0.12 US$/L versus 0.14 US$/L for {B}, and 0.17 US$/L versus 0.16 US$/L for {B + L}. This similarity could be the effect of the larger volume of ethanol produced when pentoses were also fermented to ethanol. The total volume of ethanol produced in the {C5 EtOH} cases when co-fermenting the pentoses increased by 24% for {B} and 32% for {B + L}. As a consequence, the higher revenues from electricity export, sold at 87 US$/MWh, could not compensate for the lower ethanol production, nor were they sufficient to pay off the capital cost for additional anaerobic digesters and increased CHP plant size.

An additional case was simulated to compare the 2G capital cost item in the biogas-oriented plant, and to assess both the effect of the volume of ethanol produced and the increased capital cost of additional anaerobic digestion tanks. We determined how much the capital cost item in 2G MESP would have decreased if the capital cost of the biogas-oriented plant had been the same as that of the ethanol-oriented plant. For the {C5 biogas, 10% WIS, 96 h} case this decrease would have been from 0.50 US$/L to 0.40 US$/L. However, in comparison with the {C5 EtOH, 10% WIS, 96 h} case where more ethanol is produced, the capital cost item diminished from 0.50 US$/L to 0.22 US$/L, demonstrating the predominant effect in cost reduction played by the ethanol production volume over the capital cost.

#### Enzymatic hydrolysis: residence time and WIS

During EH of the pretreated material, the sugar polymers contained in the fibre of the bagasse and leaves are converted into monomers, mainly, glucose, xylose and arabinose. The degree of conversion depends on operating conditions such as enzyme dosage, temperature, pH, WIS and residence time. In these simulations, the effects of temperature, pH and enzyme dosage were not investigated as it was assumed that optimal conditions were used (50°C, pH 4.9), and that enzymes were added at an economically reasonable dosage. The residence time and WIS loading were considered in the sensitivity analysis to establish their impact mainly on the capital cost.

The values assumed for the EH residence time were 48 hours and 96 hours to ensure a reasonable trade-off between capital cost and productivity. This led to challenging cases, which can be regarded as close to best that can be achieved, when combining pentose co-fermentation, high WIS contents and short EH residence times, for example, the {C5 EtOH, 30% WIS, 48 h} case. Reducing the EH residence time from 96 hours to 48 hours led to a decrease in the capital cost in all cases, by an amount that depended primarily on the WIS content, but also on the addition of leaves and the utilization of pentoses. Enzyme efficiency played no role as it was simply modelled as a running cost.

Extending the EH residence time to 96 hours led to an increase in the 2G MESP from 0.03 US$/L to 0.13 US$/L, representing 4% and 13% of the total 2G MESP, respectively. The longer EH residence time was also responsible for an increase in the capital cost item from 0.02 US$/L to 0.11 US$/L, compared with the 48-hour residence time.

The results regarding the increased capital cost when increasing the EH residence time from 48 hours to 96 hours can be grouped into three separate sets of values, where each set corresponds to a particular WIS content. At 30% WIS content, the increase in capital cost was in the range of 0.02 to 0.04 US$/L; at 20% WIS the range increased to 0.02 to 0.05 US$/L; and at 10% WIS the increase in the capital cost was 0.05 to 0.10 US$/L.

#### WIS

In contrast to EH residence time, WIS loading influences not only the capital cost of the EH tanks, but also the cost of the stripper columns and the energy required for distillation. High WIS loadings correspond to higher ethanol concentrations in the feed to the distillation unit, and both the cost of the distillation column and the steam required are generally lower at higher ethanol concentrations.

WIS contents above 10% led to a substantial decrease in 2G MESP in all cases. With an EH residence time of 48 hours, the average reduction in 2G MESP was 0.06 US$/L (8%) at 20% WIS and 0.09 US$/L (13%) at 30% WIS, compared to 10% WIS loading. With the longer EH residence time of 96 hours, the reduction in 2G MESP was greater: 0.11 US$/L (11%) and 0.15 US$/L (17%) at 20% WIS and 30% WIS, respectively. These results show that, in all the cases simulated, returns were diminishing by increasing the WIS from 20% WIS to 30% WIS compared to increasing from 10% WIS to 20% WIS. In fact, a 2G MESP reduction of 0.05 to 0.14 US$/L could be achieved in the former case and only 0.02 to 0.04 US$/L in the latter. Moreover, when the WIS content was increased, the reduction in the capital cost accounted for more than 68% of the reduction in the 2G MESP.

#### Addition of leaves

The addition of leaves to the integrated process is an option to increase the volume of 2G ethanol produced by increasing the plant capacity, assuming that the ethanol yield per ton cellulosic material feedstock is constant. This is in contrast to pentose co-fermentation, which leads to an increase in the amount of ethanol produced per ton raw material. The first effect of increasing the plant capacity by adding leaves will be to increase the size of the equipment and thus the total investment cost. Leaves were added in the amount of 50 wt% on dry basis of the sugarcane bagasse input to 2G process and thus increased ethanol production by 14.2% for the {C5 biogas} cases and 22.4% for the {C5 EtOH} cases. Larger equipment is generally cheaper than smaller equipment per unit volume (for example tanks) or per unit inflow (for example the filter press). For this reason, the cost per litre of ethanol produced would be lower than when using only bagasse, if the electricity opportunity cost and the cost of leaf transportation are neglected. The results of simulations showed that the capital cost per litre 2G ethanol when leaves were added was lower than when using bagasse only, for all cases simulated. The reduction in capital cost was more evident when biogas was produced from pentoses (0.03 to 0.06 US$/L or 9 to 18%). In contrast, when ethanol was produced from pentoses the reduction in capital cost was about 0.02 US$/L, regardless of the WIS content, and the relative reduction was in the range 6 to 12%.

The addition of leaves implies an increase in the electricity opportunity cost per litre of 2G ethanol, as more electricity would be generated than from bagasse only. This is the result of two main factors. The first is the difference in moisture content between leaves (15 wt%) and bagasse (50 wt%). Considering the autonomous distillery as the reference case, where leaves and/or bagasse are combusted in a CHP plant to provide steam and electricity, each ton of moisture contained in the material means a loss in electricity of about 0.25 MW_el_, due to the loss of latent heat of vaporization in the form of water vapour in the combustion off-gas. The second factor is the energy allocation for 1G ethanol production. The heat and electricity demand for 1G ethanol production is completely covered by the combustion of bagasse, and no extra energy is required to process the leaves, except in the CHP plant and handling; thus, the energy derived from leaves can be completely converted into electricity and exported. If bagasse supplemented with leaves was combusted in the autonomous distillery reference case 1G{B + L}, 124 MW_el_ could be exported, which is 1.8 times higher than the electricity generated by the combustion of bagasse only (68 MW_el_). Thus, when leaves were added, the 2G MESP was affected negatively by the higher opportunity cost of electricity, which was increased by 0.05 US$/L in the {C5 biogas} case and by 0.02 US$/L in the {C5 EtOH} case. This corresponds to 5% of the total 2G MESP, compared to cases when only bagasse was used. This small increase in opportunity cost could be affected by other factors, such as the amount of electricity obtained from the anaerobically digested stillage as well as calculation factors, such as the volume of ethanol produced.

In the {B, 10% WIS} case, for instance, the electricity opportunity cost resulting from biogas production would have been 0.25 US$/L if the pretreatment liquid fraction (68% of the initial pentoses) was anaerobically digested, and 0.12 US$/L if all waste streams were conveyed to the waste water treatment plant, allowing the highest biogas production. When biogas was not produced, the electricity opportunity cost was increased to 0.34 US$/L.

**Figure 3:**
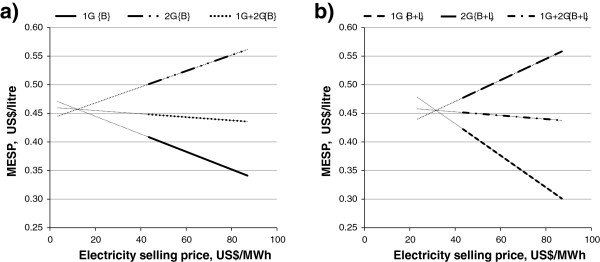
**MESP for the {C5 EtOH, 20% WIS, 96 h, 100% EHE} case.** MESPs of 1G, 2G and 1G + 2G are plotted for **(a)** the case without leaves and **(b)** with leaves addition. For case definitions see Table 1. 1G, first generation; 2G, second generation; B, bagasse; B + L, bagasse with addition of leaves; C5 EtOH, pentose fermentation to ethanol; EHE, enzymatic hydrolysis efficiency; MESP, minimum ethanol selling price; WIS, water-insoluble solids.

The electricity selling price has a considerable influence on the MESP, determining the profitability of 1G, 2G and 1G + 2G ethanol. This effect can be enhanced by the addition of leaves, as shown by the sensitivity analysis for the {C5 EtOH, 20% WIS, 96 h} case (Figure 3). 2G ethanol could almost reach the same MESP as 1G ethanol (0.46 US$/L), regardless of whether leaves are added or not. However, the electricity selling price can affect the choice of feedstock when aiming at better profitability. The addition of leaves can result in the same MESP for 1G and 2G ethanol but this requires a higher electricity selling price (32 US$/MWh) compared to when only bagasse is used (12 US$/MWh). However, as the price of electricity increases above 0.46 US$/L, the difference in MESP between 1G and 2G ethanol increases dramatically, due to electricity revenues from the 1G process and the corresponding opportunity cost in the 2G process. In any case, the overall 1G + 2G MESP was reduced as a result of increasing electricity selling price.

#### Enzymes: 100% EHE versus 250% EHE

Among the cost items determining the 2G MESP, the cost of using enzymes was the primary cost, representing 32% of the 2G MESP, which is comparable to the average capital cost in all scenarios (28 to 39%). The costs presented above are based on the budgetary price of enzymes in 2009, assuming a reasonable dosage, such as that used in laboratory experiments [54], and therefore reflect the state of the art in that year. Better enzyme cocktails are now available on the market, giving improved performance at lower cost. Therefore, the 2G MESP can be significantly reduced by using new enzymes [55]. Since the cost of enzymes was adopted as an indicator of the performance of enzymatic technology, future improvement in EHE by introducing new enzymes was modelled as a reduction in the enzyme cost. All the cases were re-evaluated assuming a more efficient enzyme cocktail, with an efficiency of 250% compared to the previous cocktail (100% EHE), or, alternatively, being 2.5 times cheaper in terms of cost. As expected, the more efficient cocktail led to a significantly lower proportion of the 2G MESP being attributed to the enzyme cost: 16%, compared with 32% previously. Moreover, the 2G MESP was reduced by 0.19 US$/L in the case of biogas production from pentoses, and by 0.11 US$/L in the case of pentose fermentation to ethanol. Since a fixed yield of 95% for EH was assumed, the WIS content, the EH residence time and the addition of leaves had no influence on the enzyme efficiency, so the enzyme cost can be regarded as an operating cost, depending purely on enzyme dosage and the volume of ethanol produced. However, experience from laboratory studies shows that the yield varies with these three variables, as does the ethanol production volume and ultimately the enzyme cost per litre of ethanol produced.

**Figure 4:**
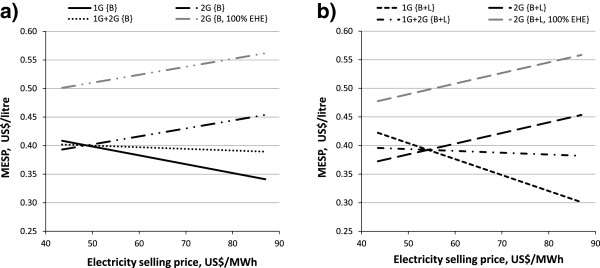
**MESP for the {C5 EtOH, 20% WIS, 96 h, 250% EHE} case.** MESPs of 1G, 2G and 1G + 2G are plotted for **(a)** the case without leaves and **(b)** with leaves addition. For case definitions see Table 1. 1G, first generation; 2G, second generation; B, bagasse; B + L, bagasse with addition of leaves; C5 EtOH, pentose fermentation to ethanol; EHE, enzymatic hydrolysis efficiency; MESP, minimum ethanol selling price; WIS, water-insoluble solids.

Besides the reduction in the 2G MESP, a 250% efficient enzyme cocktail could considerably reduce the gap between 1G MESP and 2G MESP due to electricity revenue and could also enhance the profitability of 2G ethanol, depending on the electricity selling price. Considering the {C5 EtOH, 20% WIS, 96 h, 250% EHE} cases (summarized in Table 3) at the current electricity selling price (87 US$/MWh), 2G ethanol would be competitive with 1G ethanol if 0.23 US$/L and 0.26 US$/L were provided as subsidies for bagasse {B} and bagasse with addition of leaves {B + L}, respectively. If 100% EHE enzymes were used, the subsidies required would increase by 0.10 US$/L. The use of 250% EHE enzymes was also beneficial for 2G ethanol in relation to the electricity selling price. In the sensitivity analysis shown in Figure 4 the point of equal MESP was moved from 12 US$/MWh found in Figure 3a towards higher (and more realistic) electricity selling prices, indicating that 2G ethanol could compete with 1G ethanol without subsidies at 49 US$/MWh for bagasse only and at 54 US$/MWh for bagasse supplemented with leaves. Nonetheless, the MESP at the point of equal MESP was also decreased from 46 US$/L in the {100% EHE} case in Figure 3 to about 40 US$/L in the {250% EHE} case in Figure 4, thus broadening the revenue margins. As discussed above, the addition of leaves led to greater electricity export in the 1G ethanol distillery, increasing the relative cost of 2G ethanol. It should be pointed out that below the point of equal MESP (lower electricity selling price) 2G ethanol is cheaper than 1G ethanol. The addition of leaves reduced the 2G MESP and was also beneficial for combined 1G + 2G ethanol production (see Table 3).

**Table 3:** Production and economic summary for 1G scenarios and {20% WIS, 96 h, 250% EHE} cases

				**{20% WIS, 96 h, 250% EHE}**			
		**1G**		**1G + 2G**			
**Factor**	**Unit**			**C5 biogas**		**C5 EtOH**	
		**B**	**B + L**	**B**	**B + L**	**B**	**B + L**
**Ethanol production**	L/dry ton SC^a^	274	274	387	442	478	585
**Electricity export**	MW	68	124	42	69	12	30
**Electricity export**	kWh/ton SC^b^	126	230	78	128	22	56
**Total investment cost**^**c**^	million US$	217	261	408	486	359	454
**2G MESP**	US$/BOE	-	-	162	156	93	93
**1G + 2G MESP**	US$/BOE	69	61	97	97	79	78

^a^Leaves are excluded; ^b^denotes ton of sugarcane including 70.6 wt% moisture, leaves are excluded; ^c^estimated by using Aspen Process Economic Analyzer and includes the equipment capital cost, installation, buildings, labour and contingency. For case definitions see Table 1. 1G, first generation; 2G, second generation; B, bagasse; B + L, bagasse with addition of leaves; BOE, barrel of oil equivalent; C5 biogas, biogas production from pentoses; C5 EtOH, pentose fermentation to ethanol; EHE, enzymatic hydrolysis efficiency; SC, sugarcane, WIS, water-insoluble solids.

A similar pattern of results to those presented above for 100% EHE as a function of WIS, pentose utilization and EH residence time were obtained for 250% EHE, apart from a shift in the values of the MESP.

**Figure 5:**
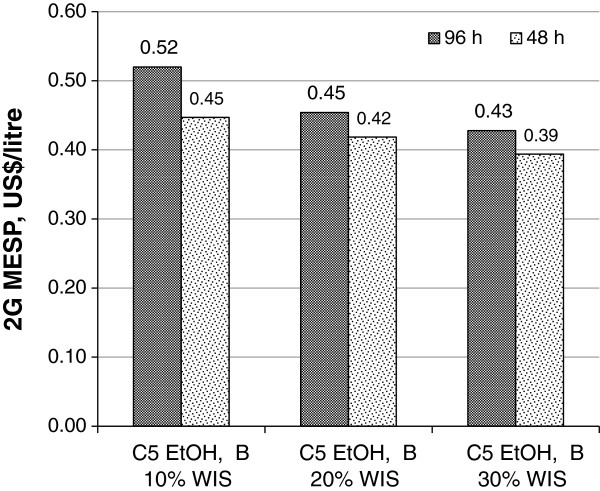
**Effect of WIS and EH residence time on 2G MESP for the {C5 EtOH, B, 250% EHE} case.** Three WIS levels (10%, 20%, 30%) and two residence times (48 hours, 96 hours) are the variables used for the comparison of 2G MESPs when ethanol is produced also from bagasse pentoses at high enzyme efficiency. For case definitions see Table 1. 2G, second generation; B, bagasse; C5 EtOH, pentose fermentation to ethanol; EH, enzymatic hydrolysis; EHE, enzymatic hydrolysis efficiency; MESP, minimum ethanol selling price; WIS, water-insoluble solids.

A particular case with 250% EHE may be relevant for the hydrolysis strategy. Considering the effect of WIS on EH residence time for the cases when bagasse was the only feedstock and the pentoses were co-fermented to ethanol {C5 EtOH, B, 250% EHE} (Figure 5), the highest reduction in 2G MESP was achieved by running EH for 96 hours at 20% WIS (0.45 US$/L), which can be compared with low WIS, that is 10% WIS (0.52 US$/L). Moreover, results also suggested that the shorter residence time and lower WIS {10% WIS, 48 h} may correspond to an equivalent 2G MESP when having high WIS and longer EH residence time.

### 1G + 2G MESP

The volume of 1G + 2G ethanol is calculated as the sum of the fixed 1G ethanol volume and the 2G ethanol volume, of which the latter varies depending on whether leaves were added and/or pentoses were fermented to ethanol. As a result, the MESP for 1G + 2G is the average of the 1G MESP and the 2G MESP weighted by the ethanol output. For this reason, the variation in 1G + 2G MESP appears to be lower than the variation in 2G MESP (Table 2). The MESP for integrated 1G + 2G ethanol production ranged from 0.41 to 0.62 US$/L, and using the 250% EHE enzymes gave a decrease of about 0.06 US$/L (11%) (Table 4). The range of values for 2G MESP was 0.50 to 1.14 US$/L, although it was slightly reduced when 250% EHE enzymes were used (0.39 to 0.95 US$/L). The effect of using the pentoses to produce ethanol was to decrease the average value of the 1G + 2G MESP by about 0.11 US$/L.

**Table 4:** 1G + 2G cost items and 1G + 2G MESP for all cases in US$/L

	**C5 biogas**						**C5 EtOH**					
**Cost items for 1G + 2G MESP**	**B**			**B + L**			**B**			**B + L**		
	**10% WIS**	**20% WIS**	**30% WIS**	**10% WIS**	**20% WIS**	**30% WIS**	**10% WIS**	**20% WIS**	**30% WIS**	**10% WIS**	**20% WIS**	**30% WIS**
Sugarcane	0.17	0.17	0.17	0.15	0.15	0.15	0.13	0.13	0.13	0.11	0.11	0.11
Leaves	0.00	0.00	0.00	0.02	0.02	0.02	0.00	0.00	0.00	0.01	0.01	0.01
Capital cost, 48 h	0.24	0.22	0.21	0.26	0.24	0.23	0.17	0.16	0.15	0.18	0.16	0.16
Capital cost, 96 h	0.27	0.23	0.22	0.30	0.25	0.24	0.19	0.17	0.16	0.20	0.18	0.17
Labour, maintenance, 48 h	0.07	0.07	0.07	0.08	0.07	0.07	0.05	0.05	0.05	0.05	0.05	0.05
Labour, maintenance, 96 h	0.08	0.07	0.07	0.09	0.08	0.07	0.06	0.05	0.05	0.06	0.05	0.05
Net electricity cost, 48 h	-0.06	-0.06	-0.06	-0.08	-0.09	-0.09	-0.02	-0.02	-0.02	-0.03	-0.03	-0.03
Net electricity cost, 96 h	-0.06	-0.06	-0.06	-0.08	-0.08	-0.08	-0.02	-0.01	-0.01	-0.03	-0.03	-0.03
Enzymes, 100% EHE	0.09	0.09	0.09	0.12	0.12	0.12	0.08	0.08	0.08	0.09	0.09	0.09
Enzymes, 250% EHE	0.04	0.04	0.04	0.05	0.05	0.05	0.03	0.03	0.03	0.04	0.04	0.04
Acid	0.01	0.01	0.01	0.02	0.02	0.02	0.01	0.01	0.01	0.01	0.01	0.01
Base	0.004	0.004	0.004	0.004	0.004	0.004	0.003	0.003	0.003	0.003	0.003	0.003
Water consumption	0.004	0.002	0.002	0.005	0.003	0.002	0.002	0.001	0.001	0.003	0.001	0.001
**1G + 2G MESP {48 h, 100% EHE}**	0.54	0.51	0.50	0.57	0.54	0.53	0.43	0.42	0.41	0.44	0.41	0.41
**1G + 2G MESP {96 h, 100% EHE}**	0.58	0.53	0.52	0.62	0.55	0.54	0.46	0.44	0.42	0.48	0.44	0.43
**1G + 2G MESP {48 h, 250% EHE}**	0.48	0.45	0.44	0.50	0.46	0.45	0.39	0.37	0.36	0.38	0.36	0.35
**1G + 2G MESP {96 h, 250% EHE}**	0.52	0.47	0.46	0.55	0.48	0.47	0.42	0.39	0.38	0.42	0.38	0.37

For case definitions see Table 1. 1G, first generation; 2G, second generation; B, bagasse; B + L, bagasse with addition of leaves; C5 biogas, biogas production from pentoses; C5 EtOH, pentose fermentation to ethanol; EHE, enzymatic hydrolysis efficiency; MESP, minimum ethanol selling price; WIS, water-insoluble solids.

The effect of EH residence time on the 1G + 2G MESP was found to be at most 0.05 US$/L higher for a residence time of 96 hours compared with 48 hours. In the {C5 biogas} cases, the WIS loading also affected the 1G + 2G MESP. A saving of 0.04 US$/L was made by increasing the WIS to 20%, and a saving of 0.07 US$/L was observed at 30% WIS. In the {C5 EtOH} cases the savings at higher WIS concentrations were reduced by about 33%.

Despite the fact that comparable values of 1G + 2G MESP were obtained with and without leaves, the addition of leaves had a minor negative impact on the 1G + 2G MESP (0.03 US$/L) when pentoses were digested to biogas. In the integrated 1G + 2G ethanol plant, the electricity export increased by between 20 and 40 kWh/ton of wet bagasse and leaves, when leaves were added, due to the increase in the amount of EH residues burned in the CHP plant, and more biogas being produced from waste streams. The addition of leaves increased the electricity revenues at most by 0.02 US$/L, but this was not always sufficient to compensate for the associated rise in capital and maintenance costs, which was at most 0.04 US$/L.

The most interesting case regarding feasibility and profitability was {C5 EtOH, 20% WIS, 96 h, 250% EHE}. When bagasse was used, the 1G + 2G MESP was 0.39 US$/L, which was very close to the 1G MESP of 0.34 US$/L. When leaves were added, the difference between the 1G + 2G MESP and the 1G MESP increased to 0.08 US$/L. However, it would not be correct to conclude that 1G ethanol is more profitable than 1G + 2G ethanol, and that bagasse would be a more profitable feedstock than bagasse and leaves simply because the former would require a smaller subsidy (0.05 US$/L) than the latter (0.08 US$/L) to reach the 1G MESP. The 2G ethanol production volume should also be taken into account in profitability analysis, as different revenues can be obtained depending on the wholesale ethanol price (WEP) at the gate, that is the price paid to the producer including the cost of raw materials, capital and operating costs, as well as the producer’s profit.

### Profitability analysis

Parameters suitable for expressing profitability are the internal rate of return (IRR), which describes the yield on investment, and the net present value (NPV), which provides a measure of the investment value. IRR and NPV were considered in the investment evaluation, as well as the main market factors such as the WEP and the electricity selling price, as both ethanol and electricity were produced in the same plant.

**Figure 6:**
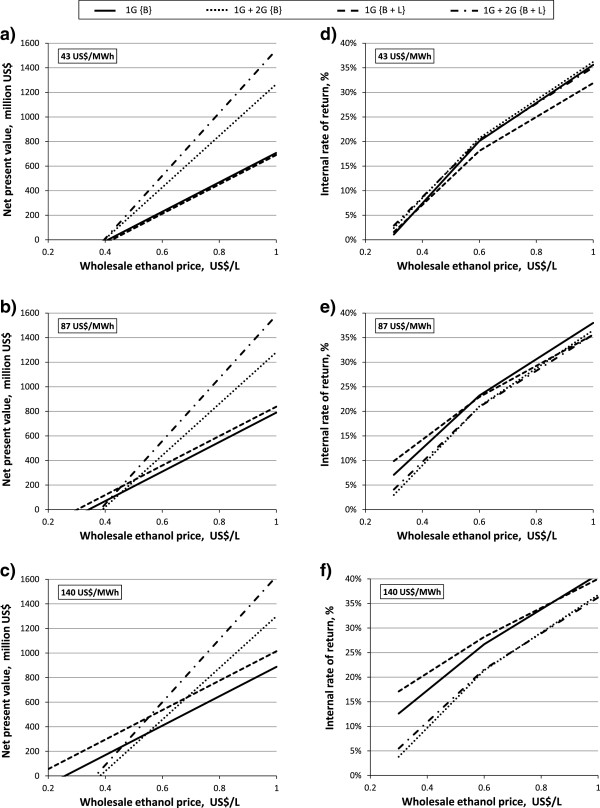
**Effect of wholesale ethanol price on NPV and IRR for the {C5 EtOH, 20% WIS, 96 h, 250% EHE} case.** Leaves addition to bagasse {B + L} and bagasse only {B} are the two feedstock used in evaluating the 1G + 2G ethanol and 1G ethanol profitability as the function of three electricity selling prices (43, 87, 140 US$/MWh). The electricity selling prices are specified in panels: 43 US$/MWh in **(a)** and **(d);** 87 US$/MWh in **(b)** and** (e);** 140 US$/MWh in **(c)** and **(f**). For case definitions see Table 1. 1G, first generation; 2G, second generation; B, bagasse; B + L, bagasse with addition of leaves; C5 EtOH, pentose fermentation to ethanol; EHE, enzymatic hydrolysis efficiency; IRR, internal rate of return; NPV, net present value; WIS, water-insoluble solids.

Three electricity selling prices were used to analyse the variation in the WEP: 43, 87 and 140 US$/MWh. According to the sensitivity analysis for the {C5 EtOH, 20% WIS, 96 h, 250% EHE} case, the NPV is usually higher for 1G + 2G ethanol than for 1G ethanol, and the addition of leaves gave the highest NPV, as can be clearly seen in Figure 6a,b,c. However, when the electricity selling price increased, the 1G ethanol plant outperformed the 1G + 2G plant, and higher values of the WEP were required to counterbalance the electricity revenues from 1G ethanol. In contrast, the IRR analysis (Figure 6d,e,f) showed that similar IRR values were obtained primarily at low electricity selling prices and the IRR curves for the 1G + 2G cases overlapped. At current electricity selling prices (87 US$/MWh), 1G ethanol could achieve higher NPV than 1G + 2G ethanol only if the WEP was below 0.52 US$/L when using bagasse and below 0.44 US$/L when using bagasse and leaves. Above these values, 1G + 2G ethanol became more profitable than 1G ethanol, despite the lower electricity export, because of the higher volume of ethanol produced. However, when the volume of ethanol decreases due to lower yields and/or to biogas production from pentoses, it may be possible that the higher electricity production in the 1G plant could be more profitable than producing 2G ethanol. The addition of leaves led to an increase in ethanol production volume and electricity export compared to bagasse without leaves, and this could be the reason why 1G + 2G ethanol from bagasse supplemented with leaves was always more profitable in terms of NPV than 1G + 2G ethanol using bagasse only. The lines representing the NPV for 1G ethanol have the same slope because the volume of ethanol produced is the same for the cases when bagasse and bagasse with leaves are combusted in the CHP plant. The offset of these two parallel lines is due to the 56 MW_el_ extra electricity produced when using bagasse and leaves, which increases the NPV by about 49 million US$. The benefits of adding leaves are more evident for 1G ethanol at low WEP due to the extra revenue from the electricity generated, while for 1G + 2G ethanol it is beneficial at WEP greater than 0.44 US$/L because of high ethanol production volume. At the lowest electricity selling price (43 US$/MWh) the volume of ethanol produced, in terms of NPV, could orient the choice towards 1G + 2G {B + L}. At the highest electricity selling price (140 US$/MWh) the exported electricity revenue from the 1G ethanol plant suggests that 1G + 2G ethanol would be the best option only if the WEP were greater than 0.55 US$/L. Such a less realistic electricity selling price was chosen to show whether 1G + 2G ethanol could be still profitable in this extreme case depending on the current market WEP.

Regarding the analysis of IRR, shown in Figure 6d,e,f, the WEP at the low electricity selling price (43 US$/MWh) has a weak effect on the IRR of the four cases compared, although the 1G + 2G ethanol scenarios seem to be slightly better than the 1G scenarios. However, the difference in IRR is already apparent at 87 US$/MWh and peaking at 140 US$/MWh. At the latter electricity selling price figure, the 1G ethanol overcomes 1G + 2G ethanol. Therefore, 1G ethanol plants could have a better yield on investment. From the investors’ point of view, the most appealing case would perhaps not be the one with highest IRR (profit yield), but the scenario with the highest NPV (profit magnitude), especially if the initial investment is high. In fact, the volume of ethanol in 2G production plays a fundamental role in overall ethanol NPV profitability, particularly when the WEP increases.

Apart from the variation in WEP, the electricity selling price is another major factor that can lead to competition and hamper 2G ethanol production, by affecting the investors’ choice based on IRR and NPV. Thus, sensitivity analysis regarding the electricity selling price was performed on the same cases using a WEP of 0.60 US$/L, which is the selling price of anhydrous ethanol averaged over the years from 2001 to 2011 in the state of São Paulo, Brazil [56].

**Figure 7:**
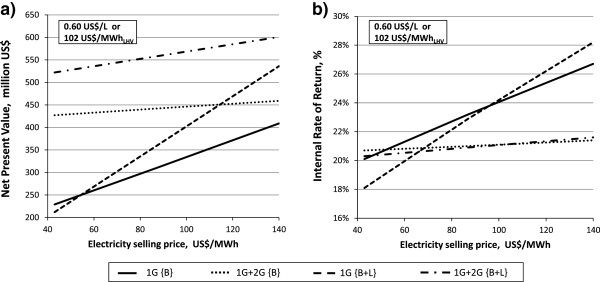
**Effect of electricity selling price on NPV and IRR for the {C5 EtOH, 20% WIS, 96 h, 250% EHE} case.** Leaves addition to bagasse {B + L} and bagasse only {B} are the two feedstock used in evaluating the 1G + 2G ethanol and 1G ethanol profitability at constant wholesale ethanol price (0.60 US$/L, corresponding to 102 US$/MWh of ethanol lower heating value, as specified in panels **(a)** and **(b)**). For case definitions see Table 1. 1G, first generation; 2G, second generation; B, bagasse; B + L, bagasse with addition of leaves; C5 EtOH, pentose fermentation to ethanol; EHE, enzymatic hydrolysis efficiency; IRR, internal rate of return; NPV, net present value; WIS, water-insoluble solids.

If NPV is used as an indicator of the highest yield on investment, rather than IRR, the 1G + 2G {B + L} case is the best, as can be seen in Figure 7a. The driver providing the highest profitability is the high ethanol volume (m^3^/dry sugarcane). The second best case is 1G + 2G {B} for an electricity selling price up to 115 US$/MWh.

As shown in Figure 7b, no single case gives the highest IRR, but it depends on the electricity selling price. The graph can be divided into three regions depending on the three drivers: ethanol production volume, low capital investment and high electricity export. When the electricity selling price is low, the greater volume of ethanol resulting from 2G production, compared with 1G, is the main driver for the high IRR up to 52 US$/MWh for 1G + 2G {B} and to 48 US$/MWh for 1G + 2G {B + L}. Then 1G {B} represents the case with highest IRR in the range 52 to 98 US$/MWh, due to the lower plant investment (217 million US$) compared to 1G {B + L} (261 million US$) despite the low electricity production (56 MW). Above 98 US$/MWh, the electricity selling price is high enough to allow high revenues from the electricity export (124 MW) produced in case 1G {B + L}.

### Feedstock

**Figure 8:**
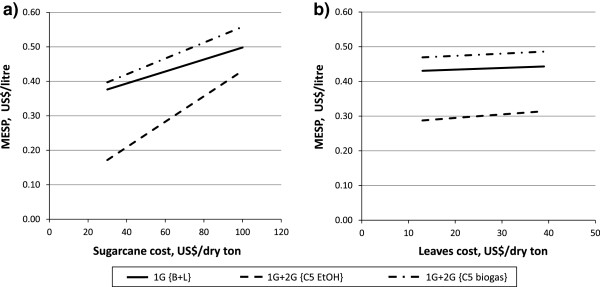
**Effect of variation in the cost of sugarcane and leaves on MESP.** The sensitivity analysis of the feedstock cost is performed on 1G + 2G MESP for the {B + L, 20% WIS, 96 h, 250% EHE} cases and on 1G MESP for the {B + L} case. For case definitions see Table 1. 1G, first generation; 2G, second generation; B + L, bagasse with addition of leaves; EHE, enzymatic hydrolysis efficiency; MESP, minimum ethanol selling price; WIS, water-insoluble solids.

Variation in the cost of feedstock had a considerable impact on operating costs and on the overall MESP of the {B + L, 20% WIS, 96 h, 250% EHE} case, due to the cost of sugarcane and, to a lesser extent, leaves. Sensitivity analysis based on feedstock prices showed the sugarcane cost to be responsible for the greatest variation in the 1G + 2G MESP, as can be seen in Figure 8. Increasing the cost of sugarcane by 50% led to increases in the MESPs of 0.06 US$/L (14%), 0.08 US$/L (17%) and 0.13 US$/L (43%) for the 1G + 2G {C5 EtOH}, 1G + 2G {biogas} and 1G cases, respectively. The effect of increasing the cost of leaves by 50% on MESP was one tenth of that with the higher sugarcane cost, because of the small amount of leaves used and the cheaper price per dry ton. For variations in the cost of sugarcane and leaves, it was noted that the increase in 1G + 2G MESP was inversely proportional to the volume of ethanol produced; thus, the highest increase in MESP corresponded to the case in which the lowest volume of ethanol was produced.

## Conclusions

The 2G ethanol production cost is affected by process options and conditions, operating costs and market prices. 2G MESP was found to be generally higher than 1G MESP although for a few of the better cases the difference was small.

The factor with the greatest effect on 2G MESP was the volume of 2G ethanol produced per ton sugarcane, rather than the input capacity of the 2G section of the plant. Thus the greatest reduction was obtained when all the sugars, including the pentoses, were co-fermented to ethanol. More efficient and profitable alternatives may be considered in future studies on biogas use, such as combustion in a combined cycle or upgrading for transportation fuel and domestic heating purposes.

The second most important mean of reducing the 2G MESP was enhancing the EHE by 2.5 times. As a result, 2G ethanol was cheaper than 1G ethanol for a wider range of electricity selling prices. The cost of EH could also be reduced, but to lesser extent, by shortening the residence time and increasing the WIS loading, both of which had a similar effect on 2G MESP reduction. The addition of sugarcane leaves had no effect on the overall 2G MESP as the gain achieved by a lower capital cost was cancelled out by a higher electricity opportunity cost per litre of 2G ethanol.

In our opinion, the {C5 EtOH, 20% WIS, 96 h, 250% EHE} cases were the most interesting for feasibility and profitability. However, the addition of leaves was found to be crucial for the profitability of 1G + 2G ethanol production in terms of NPV. The main reason for the higher revenue can be ascribed to the high volume of ethanol produced from bagasse and leaves, which also depends on conversion yields. Thus, ethanol produced in the integrated 1G + 2G plant can be more profitable than 1G ethanol from an autonomous distillery, despite the fact that the MESP for the 1G + 2G plant is greater than that for the 1G plant. However, at lower conversion yield and/or when biogas is produced from pentoses, it may be possible that the higher electricity export could improve the profitability of 1G ethanol over 1G + 2G ethanol.

## Materials and methods

The analysis of the main variables affecting the ethanol production cost in an integrated 1G + 2G plant is based on process simulations and economic evaluation of scenarios obtained by the systematic combination of the variables considered. In each case, mass and energy balances were obtained using the model implemented in Aspen Plus v7.2 (Aspen Technology, Inc, Burlington, MA, USA), and these were then used in Aspen Process Economic Analyzer v7.2 to obtain the capital cost. The overall investment cost and ethanol production cost, expressed as the MESP, were calculated in an Excel spreadsheet.

The methodology employed for this techno-economic analysis has been extensively described previously [20]. A specific flowsheet configuration (Figure 1) for an integrated 1G + 2G plant was selected from among the scenarios already investigated, and a few minor modifications were made regarding the configuration. This flowsheet model corresponds to Scenario I in the previous study, and includes all the steps in a traditional autonomous distillery (milling, clarification, evaporation, fermentation, distillation), together with the enzymatic process used to produce ethanol from lignocellulose (steam pretreatment, SHF, solid/liquid separation, distillation). Waste water treatment and the CHP plant were included with slight modifications to the previous configuration. In the reference scenarios where only 1G ethanol was produced and the lignocellulosic fractions were combusted, waste water treatment consisted of only aerobic treatment to reduce the COD. Biogas production from stillage was included in all simulations of the integrated 1G + 2G plant, and was modelled as continuous recirculation tanks instead of stirred tank reactors. The CHP plant was upgraded from a steam pressure of 65 bar to 90 bar. Stream integration in the 1G + 2G plant took place in a single distillation unit instead of in a single fermentation unit due to the possibility of employing different microorganisms for 1G and 2G ethanol, fermenting different substrates at different optimal conditions. Energy integration was implemented by heat exchange between the streams in the 1G and 2G processes. Ethanol was dehydrated by molecular sieves to 99.5 wt%. The main characteristics of the configuration modelled are given in Table 5.

**Table 5:** Process data and yield assumptions

**Parameter**	**Value**	**Unit**
Sugarcane feedstock	165	dry ton/h
Leaves:bagasse ratio added to the process	50%	dry weight
Steam pretreatment - sugar yield	95%	of theoretical
Steam pretreatment - residence time	5	minutes
Enzymatic hydrolysis - release of sugars	95%	of theoretical
Ethanol fermentation - yield from hexoses and pentoses	95%	of theoretical
Ethanol fermentation - residence time	8.2	hours
Solid/liquid separation - yield	95%	of theoretical
Methane yield from sugars, alcohols and organic acids	95%	of theoretical
COD converted to biogas	95%	of theoretical
COD conversion rate	1.0	g_COD_/L/h
Ethanol titer after distillation and dehydration	99.5%	weight
Boiler pressure	90	bar
Boiler temperature	530	°C

COD, chemical oxygen demand.

Another objective was also to determine the maximum potential of 2G ethanol production and the corresponding lowest 2G MESP. Yields close to theoretical values were chosen for the most critical steps in the 2G process. All the yields were set to 95% of the theoretical (or losses set to 5%) for steam pretreatment, EH, fermentation and solid/liquid separation, regardless of whether the operating conditions were harsh or mild, giving an overall ethanol yield of 82% from bagasse and leaves. Yields used for the 1G fermentation of sucrose were obtained from a commercial autonomous distillery [20]. The methane yield from organic acids, alcohols and sugars was also set to 95% of the theoretical, but for more recalcitrant compounds such as hydroxymethylfurfural and furfural this was reduced to 60%. Lignin and unreacted fibre were not considered to yield any biogas. The bagasse and leaves composition used as model input was determined by the CaneBioFuel project partners [57]. Sugarcane tops and stubble were removed from the trash (or straw) sample prior to the compositional analysis in order to obtain the characterization of leaves only. The variables included in the study were process options (use of pentoses and addition of leaves to bagasse), EH variables (residence time and WIS), and cost factors (enzymes, electricity, ethanol) (Table 1).

The capital cost evaluation was updated to the first quarter of 2011, as were vendor quotations for the CHP plant, the filter press and the biogas recirculation tanks. The capital cost was scaled for higher capacity with the six-tenth rule for the CHP plant and the steam dryer, while multiple pieces of equipment of the same size (the maximum size supplied by vendors) were added for steam pretreatment, filter press and anaerobic digester. Electricity is accounted for as an opportunity cost: negative for the 1G process (revenue from selling electricity produced by burning bagasse) and positive for the 2G plant (cost due to the increased energy demand compared with the 1G process alone). The indicators used for evaluation of the economic performance were the MESP for 2G ethanol (2G MESP) and for 1G + 2G ethanol (1G + 2G MESP), and the NPV and IRR on the investment. In cases where leaves were added to produce 2G ethanol, the 2G MESP was calculated using the 1G MESP corresponding to the scenario with the addition of leaves as a reference. In the sensitivity analysis, IRR and NPV were obtained as functions of the total volume of ethanol produced, and the wholesale prices of ethanol and electricity. For some interesting cases, the MESP was also expressed in terms of equivalent oil prices. A 16% refiner margin was added to the oil price to give the wholesale price of gasoline, and this was compared, on the basis of energy, to the MESP at the gate. The economic data used in the various configurations are given in Table 6.

**Table 6:** Main assumptions used for the economic calculations

**Parameter**	**Value**
IRR after tax, above inflation	10%
NPV duration	20 years
Tax rate	34%
Period of tax-deductible linear depreciation in capital cost	10 years
Plant scrap value	None
Payment of total project investment prior to start-up	12 months
Working capital (% of turnover)	20%
Financing	100% equity
Currency basis	2011 US$

IRR, internal rate of return; NPV, net present value.

## Abbreviations

1G: First generation; 2G: Second generation; 1G + 2G: Combined first and second generation; B: Bagasse; B + L: Bagasse with leaves addition; BOE: Barrel of oil equivalent; C5: Pentose sugars; C5 biogas: Biogas production from pentoses; C5 EtOH: Pentose fermentation to ethanol; CHP: Combined heat and power; COD: Chemical oxygen demand; EH: Enzymatic hydrolysis; EHE: Enzymatic hydrolysis efficiency; IRR: Internal rate of return; MESP: Minimum ethanol selling price; NPV: Net present value; SC: sugarcane; SHF: separate hydrolysis and fermentation; WEP: Wholesale ethanol price; WIS: Water-insoluble solids.

## Competing interests

The authors declare that they have no competing interests.

## Authors’ contributions

SM designed and carried out the simulations, interpreted the data and wrote the paper. OW and MG interpreted the data and revised the paper. All authors read and approved the final manuscript.

## References

[B1] The European Parliament and the Council of the European Union Directive 2003/30/EC of the European Parliament and of the Council of 8 May 2003 on the Promotion of the Use of Biofuels or Other Renewable Fuels for Transport 2003 Brussels

[B2] The European Parliament and the Council of the European Union Directive 2009/28/EC of the European Parliament and of the Council of 23 April 2009 on the Promotion of the Use of Energy from Renewable Sources 2009 Brussels

[B3] The Senate and House of Representatives of the United States of America in Congress Energy Policy Act of 2005 2005 Washington, DC [http://www.gpo.gov/fdsys/pkg/PLAW-109publ58/html/PLAW-109publ58.htm]

[B4] The Senate and House of Representatives of the United States of America in Congress Energy Independence and Security Act of 2007 2007 Washington, DC [http://www.gpo.gov/fdsys/pkg/PLAW-110publ140/html/PLAW-110publ140.htm]

[B5] Minister of Agriculture, Livestock and Food Supply, Brazil Dispõe sobre a adição de álcool etílico anidro combustível à gasolina Ordinance number 143 of 27.06.2007 2007 Brasília

[B6] Galdos M Cavalett O Seabra JEA Nogueira LAH Bonomi A Trends in global warming and human health impacts related to Brazilian sugarcane ethanol production considering black carbon emissions Appl Energy 2013 104 576 582

[B7] García CA Manzini F Environmental and economic feasibility of sugarcane ethanol for the Mexican transport sector Sol Energy 2012 86 1063 1069 10.1016/j.solener.2011.09.015

[B8] Silalertruksa T Gheewala SH Long-term bioethanol system and its implications on GHG emissions: a case study of Thailand Environ Sci Technol 2011 45 4920 4928 10.1021/es1040915 21528843

[B9] Leal MRLV Nogueira LAH Cortez LAB Land demand for ethanol production Appl Energy 2013 102 266 271

[B10] Bacovsky D Ludwiczek N Ognissanto M Wörgetter M Status of Advanced Biofuels Demonstration Facilities in 2012 Status of Advanced Biofuels Demonstration Facilities in 2012: A Report to IEA Bioenergy Task 39 2013 Ottawa, ON: IEA Bioenergy Task 39

[B11] Balan V Chiaramonti D Kumar S Review of US and EU initiatives toward development, demonstration, and commercialization of lignocellulosic biofuels Biofuels, Bioproducts and Biorefining 2013 doi:10.1002/bbb.1436

[B12] (S&T)2 Consultants Inc Second Generation Biofuels. A Review from a Market Barrier Perspective. Prepared for IEA Bioenergy Task 39. Liquid Biofuels from Biomass 2006 Delta, BC: (S&T)2 Consultants Inc

[B13] Tyner WE Taheripour F Perkis D Comparison of fixed versus variable biofuels incentives Energy Policy 2010 38 5530 5540 10.1016/j.enpol.2010.04.052

[B14] Li SZ Chan-Halbrendt C Ethanol production in China: Potential and technologies Appl Energy 2009 86 S162 S169

[B15] Bajay SV Nogueira LAH de Sousa FJR de Sousa ELL, de Carvalho Macedo I Ethanol in the Brazilian energy matrix Ethanol and Bioelectricity: Sugarcane in the Future Energy Matrix 2011 Sao Paulo: UNICA 260 308

[B16] Dias MOS Cunha MP Jesus CDF Rocha GJM Pradella JGC Rossell CEV Maciel Filho R Bonomi A Second generation ethanol in Brazil: can it compete with electricity production? Bioresour Technol 2011 102 8964 8971 10.1016/j.biortech.2011.06.098 21795041

[B17] Khatiwada D Seabra J Silveira S Walter A Power generation from sugarcane biomass - A complementary option to hydroelectricity in Nepal and Brazil Energy 2012 48 241 254 10.1016/j.energy.2012.03.015

[B18] Chandel AK da Silva SS Carvalho W Singh OV Sugarcane bagasse and leaves: Foreseeable biomass of biofuel and bio-products J Chem Technol Biotechnol 2012 87 11 20 10.1002/jctb.2742

[B19] Pandey A Soccol CR Nigam P Soccol VT Biotechnological potential of agro-industrial residues. I: Sugarcane bagasse Bioresour Technol 2000 74 69 80 10.1016/S0960-8524(99)00142-X

[B20] Macrelli S Mogensen J Zacchi G Techno-economic evaluation of 2nd generation bioethanol production from sugar cane bagasse and leaves integrated with the sugar-based ethanol process Biotechnol Biofuels 2012 5 22 10.1186/1754-6834-5-22 22502801 PMC3350453

[B21] Galbe M Zacchi G Pretreatment: The key to efficient utilization of lignocellulosic materials Biomass Bioenergy 2012 46 70 78

[B22] Franco HCJ Magalhães PSG Cavalett O Cardoso TF Braunbeck OA Bonomi A Trivelin PCO How Much Trash to Removal from Sugarcane Field to Produce Bioenergy? 2011 Campos do Jordão: 1st Brazilian BioEnergy Science and Technology Conference

[B23] CaneBioFuel WorkPackage 4: Fermentability & Process Integration [http://cordis.europa.eu/projects/227464]

[B24] CaneBioFuel WorkPackage 4: Deliverable 4.1 [http://cordis.europa.eu/projects/227464]

[B25] Rabelo SC Filho RM Costa AC A comparison between lime and alkaline hydrogen peroxide pretreatments of sugarcane bagasse for ethanol production Appl Biochem Biotechnol 2008 148 45 58 10.1007/s12010-008-8200-9 18767207

[B26] Ferreira-Leitão V Perrone CC Rodrigues J Franke APM MacRelli S Zacchi G An approach to the utilisation of CO2as impregnating agent in steam pretreatment of sugar cane bagasse and leaves for ethanol production Biotechnol Biofuels 2010 3 7 10.1186/1754-6834-3-7 20384996 PMC2861027

[B27] Geddes CC Peterson JJ Roslander C Zacchi G Mullinnix MT Shanmugam KT Ingram LO Optimizing the saccharification of sugar cane bagasse using dilute phosphoric acid followed by fungal cellulases Bioresour Technol 2010 101 1851 1857 10.1016/j.biortech.2009.09.070 19880314

[B28] Sassner P Galbe M Zacchi G Techno-economic evaluation of bioethanol production from three different lignocellulosic materials Biomass Bioenergy 2008 32 422 430 10.1016/j.biombioe.2007.10.014

[B29] Hoyer K Galbe M Zacchi G Production of fuel ethanol from softwood by simultaneous saccharification and fermentation at high dry matter content J Chem Technol Biotechnol 2009 84 570 577 10.1002/jctb.2082

[B30] Jørgensen H Vibe-Pedersen J Larsen J Felby C Liquefaction of lignocellulose at high-solids concentrations Biotechnol Bioeng 2007 96 862 870 10.1002/bit.21115 16865734

[B31] Tengborg C Galbe M Zacchi G Influence of enzyme loading and physical parameters on the enzymatic hydrolysis of steam-pretreated softwood Biotechnol Prog 2001 17 110 117 10.1021/bp000145+ 11170488

[B32] Rudolf A Baudel H Zacchi G Hahn-Hägerdal B Lidén G Simultaneous saccharification and fermentation of steam-pretreated bagasse using Saccharomyces cerevisiae TMB3400 and Pichia stipitis CBS6054 Biotechnol Bioeng 2008 99 783 790 10.1002/bit.21636 17787015

[B33] Carrasco C Baudel HM Sendelius J Modig T Roslander C Galbe M Hahn-Hägerdal B Zacchi G Lidén G SO2-catalyzed steam pretreatment and fermentation of enzymatically hydrolyzed sugarcane bagasse Enzym Microb Technol 2010 46 64 73 10.1016/j.enzmictec.2009.10.016

[B34] Parachin NS Bergdahl B van Niel EWJ Gorwa-Grauslund MF Kinetic modelling reveals current limitations in the production of ethanol from xylose by recombinant Saccharomyces cerevisiae Metab Eng 2011 13 508 517 10.1016/j.ymben.2011.05.005 21642010

[B35] Ensinas AV Nebra SA Lozano MA Serra LM Analysis of process steam demand reduction and electricity generation in sugar and ethanol production from sugarcane Energy Convers Manag 2007 48 2978 2987 10.1016/j.enconman.2007.06.038

[B36] Wingren A Galbe M Zacchi G Energy considerations for a SSF-based softwood ethanol plant Bioresour Technol 2008 99 2121 2131 10.1016/j.biortech.2007.05.058 17900894

[B37] Ensinas AV Nebra SA Lozano MA Serra L Design of evaporation systems and heaters networks in sugar cane factories using a thermoeconomic optimization procedure Int J Thermodyn 2007 10 97 105

[B38] Sassner P Zacchi G Integration options for high energy efficiency and improved economics in a wood-to-ethanol process Biotechnol Biofuels 2008 1 4 10.1186/1754-6834-1-4 18471311 PMC2375869

[B39] Dias MOS Da Cunha MP MacIel Filho R Bonomi A Jesus CDF Rossell CEV Simulation of integrated first and second generation bioethanol production from sugarcane: comparison between different biomass pretreatment methods J Ind Microbiol Biotechnol 2011 38 955 966 10.1007/s10295-010-0867-6 20838849

[B40] Furlan FF Costa CBB Fonseca GDC Soares RDP Secchi AR Cruz AJGD Giordano RDC Assessing the production of first and second generation bioethanol from sugarcane through the integration of global optimization and process detailed modeling Comput Chem Eng 2012 43 1 9

[B41] Laser M Schulman D Allen SG Lichwa J Antal MJ Jr Lynd LR A comparison of liquid hot water and steam pretreatments of sugar cane bagasse for bioconversion to ethanol Bioresour Technol 2002 81 33 44 10.1016/S0960-8524(01)00103-1 11708754

[B42] Diedericks D van Rensburg E Görgens JF Enhancing sugar recovery from sugarcane bagasse by kinetic analysis of a two-step dilute acid pretreatment process Biomass Bioenergy 2013 57 149 160

[B43] Pitarelo AP Da Silva TA Peralta-Zamora PG Ramos LP Effect of moisture content in the steam treatment and enzymatic hydrolysis of sugarcane bagasse Quím Nova 2012 35 1502 1509

[B44] Rocha GJM Martín C da Silva VFN Gómez EO Gonçalves AR Mass balance of pilot-scale pretreatment of sugarcane bagasse by steam explosion followed by alkaline delignification Bioresour Technol 2012 111 447 452 22391588 10.1016/j.biortech.2012.02.005

[B45] Cannella D Jørgensen H Do new cellulolytic enzyme preparations affect the industrial strategies for high solids lignocellulosic ethanol production? Biotechnol Bioeng 2014 111 59 68 10.1002/bit.25098 24022674

[B46] Benjamin Y Cheng H Görgens JF Evaluation of bagasse from different varieties of sugarcane by dilute acid pretreatment and enzymatic hydrolysis Ind Crop Prod 2013 51 7 18

[B47] Wingren A Galbe M Zacchi G Techno-economic evaluation of producing ethanol from softwood: Comparison of SSF and SHF and identification of bottlenecks Biotechnol Prog 2003 19 1109 1117 12892470 10.1021/bp0340180

[B48] Olofsson K Sibbesen O Andersen TV Rønnow B Rapid xylose and glucose fermentation by engineered S. cerevisiae for commercial production of cellulosic ethanol In poster presented at conference Advanced Biofuels in a Biorefinery Approach, Copenhagen; 2012. [http://www.terranol.com/downloads/Advanced_Biofuels_in_a_Biorefinery_Approach_in_Copenhagen_2012_Terranol_Poster.pdf]

[B49] Demeke MM Dumortier F Li Y Broeckx T Foulquié-Moreno MR Thevelein JM Combining inhibitor tolerance and D-xylose fermentation in industrial Saccharomyces cerevisiae for efficient lignocellulose-based bioethanol production Biotechnol Biofuels 2013 6 120 10.1186/1754-6834-6-120 23971950 PMC3765968

[B50] España-Gamboa EI Mijangos-Cortés JO Hernández-Zárate G Maldonado JAD Alzate-Gaviria LM Methane production by treating vinasses from hydrous ethanol using a modified UASB reactor Biotechnol Biofuels 2012 5 82 10.1186/1754-6834-5-82 23167984 PMC3538563

[B51] Rabelo SC Carrere H Maciel Filho R Costa AC Production of bioethanol, methane and heat from sugarcane bagasse in a biorefinery concept Bioresour Technol 2011 102 7887 7895 10.1016/j.biortech.2011.05.081 21689929

[B52] Molina F Ruiz-Filippi G García C Roca E Lema JM Winery effluent treatment at an anaerobic hybrid USBF pilot plant under normal and abnormal operation Water Sci Technol 2007 56 25 31 17849974 10.2166/wst.2007.468

[B53] Sanchez Riera F Cordoba P Sineriz F Use of the UASB reactor for the anaerobic treatment of stillage from sugar cane molasses Biotechnol Bioeng 1985 27 1710 1716 10.1002/bit.260271212 18553633

[B54] Canebiofuel Task 3.4. [http://cordis.europa.eu/projects/227464]

[B55] Novozymes Advanced Biofuels Becoming Reality with Novozymes’ New Enzyme Technology 2012 Bagsværd: Novozymes [http://www.novozymes.com/en/news/news-archive/Pages/Advanced-biofuels-becoming-reality-with-Novozymes-new-enzyme-technology.aspx]

[B56] CEPEA Ethanol price. [http://www.cepea.esalq.usp.br]

[B57] CaneBioFuel Compositional analysis [http://cordis.europa.eu/projects/227464]

